# Levels of albuminuria and risk of developing macroalbuminuria in type 2 diabetes: historical cohort study

**DOI:** 10.1038/srep26380

**Published:** 2016-05-23

**Authors:** Shoma Chida, Yoshikuni Fujita, Akifumi Ogawa, Akinori Hayashi, Raishi Ichikawa, Yuji Kamata, Akihiro Takeuchi, Koji Takano, Masayoshi Shichiri

**Affiliations:** 1 Department of Endocrinology, Diabetes and Metabolism, Kitasato University School of Medicine, Kanagawa 252-0374, Japan; 2 Department of Medical Informatics, School of Allied Health Sciences, Kitasato University, Kanagawa, Japan

## Abstract

Although increased urinary albumin excretion may increase the risk of adverse renal outcomes in patients with diabetes, it remains unclear whether microalbuminuria is associated with a higher incidence of macroalbuminuria in the absence of non-diabetic kidney events that frequently develop during the long-term course of type 2 diabetes. This historical cohort study included patients with type 2 diabetes, spot urine albumin:creatinine ratio (ACR) <300 mg/gCr and normal serum creatinine concentrations treated between August 1988 and April 2015. Patients with any evidence suggesting non-diabetic kidney diseases at baseline were excluded. Over a median follow-up of 50 months, 70 of the 1760 included patients developed macroalbuminuria. Twenty-one of these patients were diagnosed with non-diabetic renal events. The five-year cumulative incidence of macroalbuminuria in patients with ACRs of 0–7.5 mg/gCr, 7.5–30 mg/gCr, 30–150 mg/gCr, and 150–300 mg/gCr were 0%, 0.53%, 3.5%, and 36.0%, respectively, with significant differences between each pair of ACR categories. In type 2 diabetes, higher urinary ACR, even within a level of normoalbuminuria, was associated with a greater incidence of macroalbuminuria when non-diabetic renal events were excluded. These results conflict with findings suggesting that microalbuminuria is a poor indicator for the progression of diabetic nephropathy.

Microalbuminuria significantly increases the relative risk of development of diabetic nephropathy^1^,^2^,^3^ and is a risk factor for adverse cardiovascular outcomes^1^,^4^,^5^,^6^. European and American guidelines therefore recommend that diabetic patients be annually tested for albuminuria^7^,^8^, whereas Japanese guidelines encourage more frequent measurements, which has become common practice in Japan^9^. However, the accuracy of albuminuria in predicting progression to overt diabetic nephropathy and to end-stage kidney disease is unclear^10^. Microalbuminuria, defined as 30–300 mg albumin/day or 30–300 mg albumin/g creatinine (Cr) excreted in the urine, often regresses to normoalbuminuria (<30 mg/gCr) in type 1 diabetes patients, independent of renin-angiotensin blockade^11^,^12^. In contrast, regression may be associated with non-glomerular complications^13^. The development of advanced chronic kidney disease in some diabetic patients with new onset microalbuminuria may not require progression to proteinuria^14^,^15^,^16^. Some diabetic patients with chronic kidney disease show normoalbuminuria and absence of retinopathy^10^,^17^. Further, kidney biopsy specimens from patients with diabetes often reveal primary kidney diseases not attributable to diabetes^18^, whereas a significant number of type 2 diabetes patients with biopsy-proven diabetic nephropathy are normoalbuminuric^19^. Levels of urinary albumin excretion, even within the “normal” range, have been associated with an increased risk of cardiovascular diseases^5^,^20^,^21^ and a slight, but significantly higher, decline in estimated glomerular filtration rate (eGFR)^22^. The upper limit of “normoalbuminuria” has been defined as the 95th percentile of albumin excretion rate in ‘normal’ individuals^23^,^24^. The ability of this cut-off point to reliably assess the risk of nephropathy development remains to be determined. Despite all these confounding factors that reduce the biomarker value, microalbuminuria still remains the most well-studied biomarker predicting the future development of clinical nephropathy^25^,^26^. Because type 2 diabetes patients often develop kidney injury unrelated to diabetic nephropathy over the long-term, little is known about the contribution of these non-diabetic factors to the development of macroalbuminuria and the decline of renal function. Thus, the role of microalbuminuria as a marker should be rigorously assessed, after carefully excluding patients with non-diabetic renal events.

This study evaluated whether the risk of incident macroalbuminuria was related to the levels of albuminuria in a historical cohort of type 2 diabetic patients with complete medical records throughout their entire follow-up periods. Since 1988, routine follow-up protocols for diabetic patients covered by the universal health insurance system in Japan have included routine quarterly measurements of albuminuria. Complete records of automated urine analysis and subsequent fluorescent laser flow cytometry analysis of urine sediments provide important clues in detecting and diagnosing non-diabetic kidney diseases and provide information over time, determining whether incident macroalbuminuria or reduced renal function was caused by non-diabetic kidney injury.

## Results

### Participant characteristics

Between August 1988 and February 2013, 2648 diabetic patients aged ≥18 years underwent an initial diabetes scrutiny, were clinically diagnosed with type 2 diabetes and had a normal serum creatinine concentration and urinary albumin-creatinine ratio (ACR) <300 mg/gCr on repeated measurements. Patients with characteristics suggesting the near future development of non-diabetic causes of renal diseases were excluded. These include 226 obese patients (BMI >30 kg/m^2^) and 662 with persistent haematuria, repeated detection of a variety of urinary casts, and/or other definite evidence of primary non-diabetic renal diseases. Thus, a total of 1760 type 2 diabetic patients was included in our analysis. These patients contributed an overall 8345 person-years of observation. The median age of the cohort at baseline was 62 years, and 39.7% were women.

### Overall incidence of macroalbuminuria after excluding non-diabetic kidney events

After a median follow-up of 50 months (maximum, 317 months; interquartile range 19–90 months), 49 of 1760 patients (2.8%) developed macroalbuminuria without showing any evidence of non-diabetic causes of renal diseases, at an overall rate of 0.59 cases per 100 person-years. Twenty-one patients developed macroalbuminuria simultaneously with non-diabetic kidney events and were therefore separately analysed from the group with incident macroalbuminuria. Patients with non-diabetic kidney events included (1) nine with primary glomerular diseases, (2) four with drug-induced kidney injuries (one due to an anti-rheumatic agent, two caused by anti-cancer reagents and one resulting from multidrug treatment following a cerebrovascular accident), (3) one with cardiorenal syndrome associated with severe heart failure, (4) three with kidney injury associated with cancer development, (5) one with infectious kidney disease, (6) one with obstructive kidney disease, (7) one with malignant uncontrollable hypertension, and (8) one with lupus nephritis. Eight patients showed persistent elevation of serum creatinine before developing macroalbuminuria. Five of these eight patients were diagnosed with elevated creatinine unrelated to diabetic renal disease (three caused by diuretics, one by anticancer drugs and one associated with an abdominal aortic aneurysm). The causes of elevated serum creatinine concentrations in the remaining three patients could not be determined.

### Predictive performance of albuminuria

**Figure 1:**
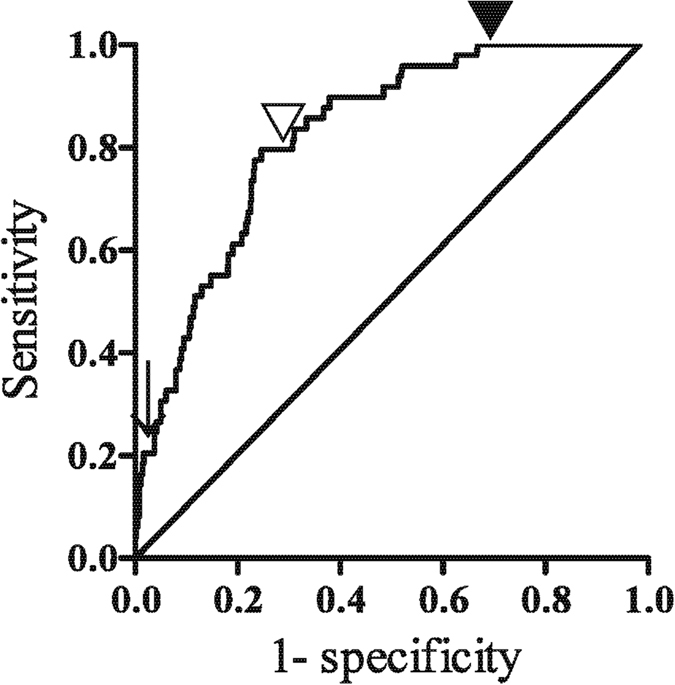
Receiver operating characteristic (ROC) curve for predicting incidence of macroalbuminuria unrelated to non-diabetic renal events by measurement of ACR. ROC was performed for baseline ACR. The area under the curve for ACR was 0.8249. Setting the ACR cut-off at 7.5 mg/gCr (▼) resulted in a sensitivity of 100% and a specificity of 31%. Setting the ACR cut-off at 30 mg/gCr (▽) resulted in a sensitivity of 80% and a specificity of 72%. Setting the ACR cutoff at 150 mg/gCr (↓) resulted in a sensitivity of 20% and a specificity of 98%.

Receiver operating characteristic (ROC) curve analysis was used to assess the performance of baseline ACR in predicting the incidence of macroalbuminuria, after excluding patients with macroalbuminuria due to non-diabetic causes (Fig. 1). The area under the ROC curve for baseline ACR was 0.82 (95% confidence interval (CI) 0.777–0.873, p < 0.0001). An ACR cut-off point of 30 mg/gCr had both optimal sensitivity (80%) and specificity (72%). The cut-off point for maximal (100%) sensitivity was 7.5 mg/gCr, which had a specificity of 31%; whereas the cutoff point for maximum specificity (98%) was 150 mg/gCr, which had a sensitivity of 20%.

### Rates of macroalbuminuria relative to ACR categories of diabetic nephropathy

**Figure 2:**
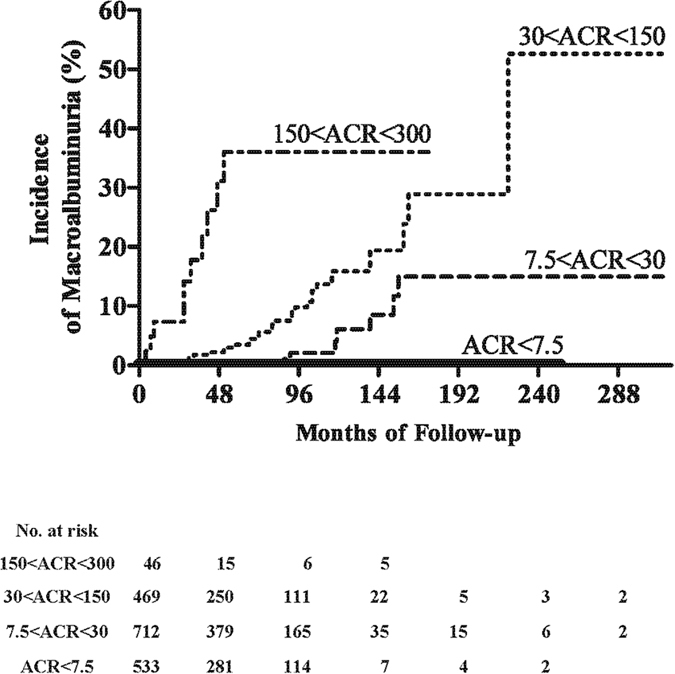
Kaplan-Meier analysis of progression to macroalbuminuria unrelated to non-diabetic renal disease in patients with type 2 diabetes. ACR denotes urinary albumin-creatinine ratio expressed as mg albumin/g creatinine. The baseline urinary ACR was defined as the mean of the first two consecutive measurements. Macroalbuminuria was defined as a urinary ACR >300 mg/gCr for at least 6 months. Subjects were censored at their death or withdrawal date.

The above ROC analysis led to a split in baseline ACR into four subgroups: <7.5, 7.5–30, 30–150, and 150–300 mg/gCr. The baseline demographic, clinical, and biochemical characteristics of these four groups, as well as their medications, are shown in Table 1. None of the 533 patients with ACR <7.5 mg/gCr developed macroalbuminuria during the entire observation period, compared with 10 (1.4%) of 712 patients with ACR 7.5–30 mg/gCr, 29 (6.2%) of 469 patients with 30–150 mg/gCr, and 10 of 46 patients (21.7%) with ACR 150–300 mg/gCr. The annual incidences of macroalbuminuria in these four groups were 0.00, 0.28, 1.27, and 6.03 per 100 person years, respectively. Kaplan-Meier analysis showed that higher baseline ACR was associated with a significantly higher cumulative incidence of macroalbuminuria (Fig. 2).

Kaplan-Meier analyses were performed using several different cut-off points to avoid the assay variability contributing to the results: (i) 0–7.5, 7.5–30, 30–60, and 60–225 mg/gCr, (ii) 0–7.5, 7.5–30, 30–130, and 130–240 mg/gCr, and (iii) 0–100, and 100–225 mg/gCr. These analyses also revealed that higher baseline albuminuria resulted in higher incidence of macroalbuminuria with significant differences between each pair of ACR categories.

### Rates of macroalbuminuria relative to ACR categories in patients with non-diabetic renal diseases

**Figure 3:**
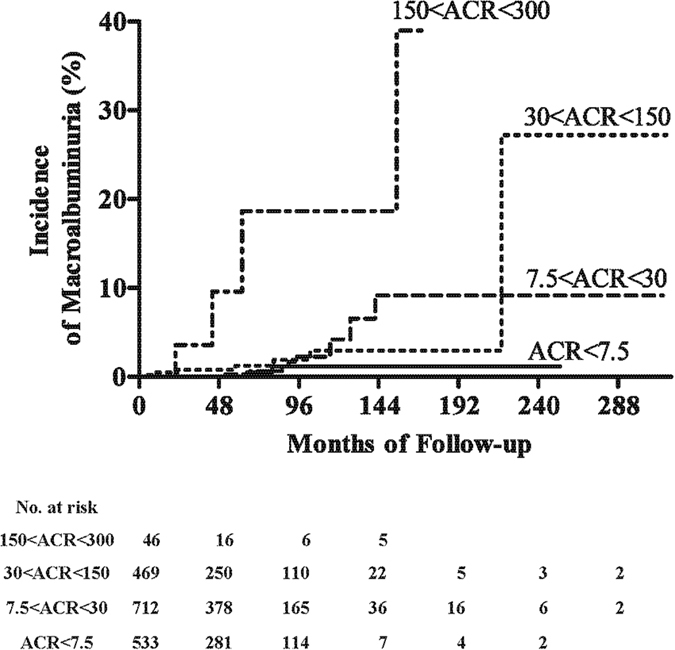
Kaplan-Meier analysis of progression to macroalbuminuria caused by non-diabetic renal diseases in patients with type 2 diabetes. ACR denotes urinary albumin-creatinine ratio expressed as mg albumin/g creatinine. The baseline urinary ACR was defined as the mean of the first two consecutive measurements. Macroalbuminuria was defined as a urinary ACR >300 mg/gCr for at least 6 months. Subjects were censored at their death or withdrawal date.

The incidence rates of macroalbuminuria clearly induced by non-diabetic kidney events were compared in the four subgroups of patients categorized by ACR. Two (0.38%) of the 533 patients with ACR <7.5 mg/gCr, eight (1.1%) of the 712 with ACR 7.5–30 mg/gCr, seven (1.5%) of the 469 with ACR 30–150 mg/gCr, and four (8.7%) of the 46 with ACR 150–300 mg/gCr developed macroalbuminuria associated with non-diabetic kidney injury during the observation period. The annual incidences of macroalbuminuria in these four groups were 0.08, 0.23, 0.31, and 2.45 per 100 person years, respectively. Kaplan-Meier analysis showed that patients in the highest ACR category (150–300 mg/gCr) experienced non-diabetic kidney events significantly more frequently than patients in the remaining three categories (p < 0.0001 each) (Fig. 3). However, there were no significant differences between pairs of the other three categories, suggesting that ACR was less predictive of macroalbuminuria due to non-diabetic than diabetic events.

### Glycosylated haemoglobin control

**Figure 4:**
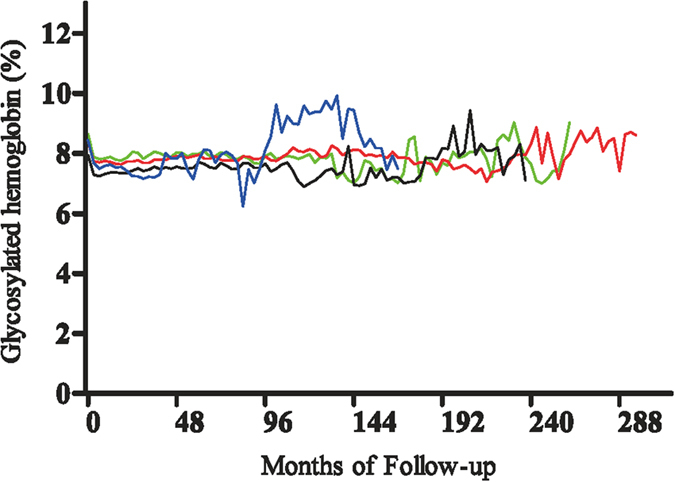
Changes in average glycosylated haemoglobin concentrations in the four baseline ACR categories. Glycosylated haemoglobin concentrations of patients in each of the four ACR categories were averaged and plotted: <7.5 mg/gCr (black line), 7.5–30 mg/gCr (red line), 30–150 mg/gCr (green line), 150–300 mg/gCr (blue line).

Sequential changes in average HbA1c levels for patients in the four ACR categories are shown in Fig. 4. Mean ± SD baseline HbA1c concentrations were significantly lower in patients with ACR <7.5 mg/gCr (7.9 ± 1.7%) than in patients with ACR 7.5–30 mg/gCr (8.4 ± 1.8%, P < 0.0001), 30–150 mg/gCr (8.6 ± 1.8%, P < 0.0001) and 150–300 mg/gCr (8.5 ± 2.4%, P = 0.048). HbA1c concentrations remained significantly lower at 48 months in patients with ACR <7.5 mg/gCr (7.6 ± 1.2%) than in patients with ACR 7.5–30 mg/gCr (7.9 ± 1.2%, P = 0.0007) and 30–150 mg/gCr (8.0 ± 1.3%, P = 0.0001), but these differences disappeared after 96 months.

## Discussion

This study showed that baseline ACR was associated with an increased incidence of macroalbuminuria in type 2 diabetic patients lacking potentially preexisting non-diabetic kidney diseases. Patients with baseline ACR of 150–300 mg/gCr had a significantly higher incidence of macroalbuminuria than patients in any of the other ACR categories, regardless of the cause of macroalbuminuria. Because elevated ACR in diabetic patients is associated with increased risks of all-cause mortality and cardiovascular events^5^,^20^,^21^, these patients are more likely to experience non-diabetic renal injury events. After excluding the 21 patients with non-diabetic renal events from the 70 with macroalbuminuria, we found that an elevated risk of nephropathy progression was more closely associated with baseline ACR category. This was the case even when comparing individuals with higher (ACR 7.5–30 mg/gCr) and lower (ACR <7.5 mg/gCr) levels of normoalbuminuria. All patients in the lowest ACR category who reached the macroalbuminuria end point showed clear-cut evidence of renal injury unrelated to diabetic nephropathy. Therefore, patients with baseline ACR <7.5 mg/gCr and without any preexisting renal disease were at negligible risk for progression to diabetic nephropathy. In contrast, patients with slightly elevated albuminuria (ACR 7.5–30 mg/gCr) were not completely free from the risk of developing macroalbuminuria. When incident macroalbuminuria due to non-diabetic causes of kidney injury was included in the current analysis, the only ACR cut-off point discriminating between neighbouring ACR categories was 150 mg/gCr (data not shown). Thus, despite the current belief that albuminuria is insensitive in identifying patients at risk of progression of diabetic nephropathy, the results of this study found that this parameter was predictive of the development of macroalbuminuria unrelated to non-diabetic renal events.

In initial enrolment of patients into this historical cohort study, we found that, of the 2648 type 2 diabetic patients with normal serum creatinine and baseline ACR <300 mg/gCr, 888 had been previously diagnosed with or potentially had non-diabetic renal disease and were therefore excluded. Our exclusion criteria included obesity, persistent haematuria, and the presence of a variety of urinary casts. We hypothesized that patients with underlying kidney diseases would progress to macroalbuminuria, irrespective of the accompanying diabetic nephropathy. However, except for limited prospective renal biopsy trials in patients with type 1 diabetes^27^,^28^,^29^, almost all previous studies evaluating microalbuminuria as a biomarker did not exclude patients with preexisting primary kidney disease or those who experienced non-diabetic renal events. Our centre has maintained the medical records of all diabetic patients seeking medical care in the Department of Endocrinology, Diabetes and Metabolism since 1971, and their detailed clinical information is available for retrospective analyses. Our follow-up protocols include automated urine analysis and subsequent urine fluorescent flow cytometry analysis using UF-1000i, or its predecessor, UF-100i, which have been regarded as using the most accurate and efficient strategies to perform urinalysis^30^,^31^,^32^,^33^,^34^,^35^. UF-1000i can determine the morphology of urinary cells, casts, crystals, bacteria, and other particles by utilising separate channels combined with fluorescent staining techniques, while simultaneously determining urinary red cell morphology to quantitatively analyse dysmorphic red cells, a technology used world-wide to diagnose haematuria of glomerular origin^36^,^37^,^38^. All significant findings were confirmed by phase-contrast microscopic examinations. This process has been used in the long-term detection of non-diabetic causes of kidney injury in patients with type 2 diabetes. All urinary test results, as well as all further outcomes, have been recorded in our electronic database, enabling the exclusion of patients with preexisting non-diabetic renal diseases and the assessment of the development of non-diagnostic kidney events during the entire follow-up period.

ACR is not elevated in substantial proportions of patients with types 1 and 2 diabetes with decreased GFR^14^,^39^,^40^,^41^,^42^. Microalbuminuria in patients with type 1 diabetes can progress to more advanced renal disease without the development of overt proteinuria^16^. It remains unclear whether renal function decline in the absence of macroalbuminuria is caused by diabetic kidney changes *per se* or by non-diabetic renal injury. In the present study, eight of the 1760 patients with normal baseline creatinine levels reached the preset serum creatinine endpoint. Five of these eight patients presented clear-cut evidence of non-diabetic renal diseases at the time of serum creatinine elevation. Our previous small follow-up study of type 2 diabetic patients excluded patients with any evidence suggestive of non-diabetic renal disease in order to recruit patients with early diabetic nephropathy alone^18^. Renal biopsy showed that one fourth of these patients clinically diagnosed with diabetic nephropathy had primary glomerular diseases, with some also having electron microscopic evidence of diabetic changes. The remaining three fourths of these patients showed no evidence of nondiabetic renal glomerular or tubular/interstitial changes. These findings suggest that, despite efforts to exclude patients with indications of non-diabetic renal disease, our cohort may still have included a significant number of patients with baseline non-diabetic renal diseases and that these latter conditions may have been responsible for macroalbuminuria and/or elevated serum creatinine in these patients.

Because of our attempt to exclude preexisting primary glomerular diseases and obesity-associated nephropathy, the current cohort does not include any patients who presented with persistent haematuria or obesity. However, haematuria can occur in diabetic kidney disease, and obesity may be common among type 2 diabetic patients while many obese people do not necessarily develop obesity-associated nephropathy. Thus, the current results do not represent exact incidence of macroalbuminuria in diabetic populations associated with these conditions. Another limitation of the current study is that the automated urinalysis and urine microscopy do not always detect the underlying glomerular diseases and thus is not an exact surrogate for renal biopsy findings. Despite these limitations, our attempts to carefully differentiate kidney diseases appeared to have detected significant number of non-diabetic kidney events within the enrolled diabetic population.

In conclusion, higher urinary ACR levels in adults with type 2 diabetes were associated with a greater incidence of macroalbuminuria, after excluding patients with clear-cut evidence of non-diabetic kidney diseases. Urinary ACR was a more reliable predictor of future progression of diabetic nephropathy than currently thought, although type 2 diabetic patients often develop non-diabetic kidney injury events over the long-term.

## Methods

### Identification of the Study Cohort

This historical cohort study involved the complete medical records of diabetic patients who had been regularly followed by diabetologists every 1–3 months at the Department of Endocrinology, Diabetes and Metabolism of Kitasato University Hospital (Kanagawa, Japan), a tertiary care centre that serves the densely populated southwestern Tokyo metropolitan area, containing approximately 1 million persons. Patients were included if they were aged ≥18 years, were clinically diagnosed with type 2 diabetes between August 1988 and February 2013, and showed ACR <300 mg/gCr, a normal serum Cr concentration (0.6 to 1.1 mg/dl [53 to 97 μmol/L] for men and 0.4 to 0.8 mg/dl [35 to 71 μmol/L] for women) and absence of any evidence suggesting the possibility of a non-diabetic renal disease. Type 2 diabetes was diagnosed according to the criteria of the Japan Diabetes Society; patients with anti-glutamic acid decarboxylase autoantibody concentrations >1.5 U/ml or serum C-peptide concentrations <0.5 ng/ml were excluded, as were pregnant women. Non-diabetic renal diseases include primary glomerular diseases, drug-induced nephropathy, obesity-related nephropathy, polycystic kidney, infectious diseases, reflux nephropathy and nephrolithiasis. Also excluded from analysis were patients persistently showing urinary casts and/or haematuria to avoid the potential presence/development of primary glomerular diseases. In addition, to avoid obesity-related nephropathy, patients with a body-mass index (BMI) >30 kg/m^2^, corresponding to the 97^th^ percentile of the entire national adult population in Japan^43^, were excluded.

### Ethics Statement

The ethics committees of Kitasato University Hospital approved this study protocol and waived the requirement for informed consent due to the retrospective nature of this study. The methods were carried out in accordance with the approved guidelines.

### Procedures, Measurements, and Outcome

Patients initially underwent chest X rays, electrocardiography, and abdominal ultrasonography. Concentrations of glycated albumin, anti-GAD antibody, serum and urinary C-peptides were measured. Cervical arterial ultrasonography was performed, and ankle-brachial blood pressure index and/or brachial ankle pulse wave velocity were measured. Routine tests performed during regular visits included a clinical examination, assessment of diet and any possible adverse reactions to prescribed medicines, blood pressure, body weight, urinalysis and urinary sediment by automated analysers, serum chemistry, complete blood count, and HbA1c concentrations. ACR was measured every 3 months. Patients showing any signs or symptoms suggestive of development of other medical conditions were assessed using additional diagnostic tests and referred to the applicable clinical department if required. Adherence to dietary therapy was assessed and dietary counselling performed by nutritionists. Poorer glycaemic control was treated according to then-current Japan Diabetes Society guidelines. Additional tests were performed on patients with unexplainable exacerbation of glycaemic control. Some patients required short-term hospitalisation for scrutiny and diabetic control. Patients underwent regular ophthalmological tests. Elevated blood pressure and dyslipidaemia were assessed and treated according to the guidelines of The Japanese Society of Hypertension and The Japan Atherosclerosis Society, respectively. Smokers were told to quit smoking. All laboratory tests of urine and blood were performed at either our central laboratory or SRL, Inc. (Tokyo, Japan). All patient management protocols conformed to routine diabetic patient management regimens covered by the Japanese universal health insurance system.

Urinary albumin concentrations were measured by immunonephelometry using an automated system Hitachi 7600 (Hitachi, Ltd., Tokyo, Japan) or JCA-BM 8040 (JEOL Ltd., Tokyo, Japan). Serum creatinine was determined with Jaffe’s reaction method during 1988–1995 and then changed to the automated enzymatic method. Glycosylated haemoglobin was measured by high-performance liquid chromatography using HLC-723G8 (Tosoh Co., Tokyo, Japan), C-peptide concentrations by a Lumipulse Presto C-peptide commercial kit (Fujirebio Co., Tokyo, Japan), and anti-GAD by a radioimmunoassay using a WALLAC 1460 gamma counter (SRL, Inc., Tokyo, Japan). Routine urinalysis was performed on a clinical lab II (Clinitek 200; Eimus, Siemens Healthcare Diagnostics Co., Tokyo, Japan), a SuperAution Analyzer II (Arkray Co., Kyoto, Japan), US-3100, US-2100 R, US-3100 R or US-3100 R plus groups (Eiken Chemical Co. Ltd., Tokyo, Japan) in chronological order. All urine specimens with protein concentrations of 2+ or higher and/or sediments containing no fewer than 5 urinary red or white blood cells per high powered field were subjected to automated morphological examination using a laser-based fluorescent flow cytometer, UF-1000i (Sysmex Co., Hyogo, Japan) to analyse urine particles^33^, including dysmorphic haematuria^36^,^37^,^38^. All urine specimens showing significant findings were further reconfirmed by phase-contrast microscopic examinations. Baseline urinary ACR was defined as the mean of the first two consecutive urine samples. The primary efficacy measure was the time from the first baseline measurement to the detection of macroalbuminuria, defined as urinary ACR >300 mg/gCr or serum creatinine exceeding 1.5 mg/dl for men and 1.3 mg/dl for women for at least 6 months. Patients more than 6 months late for a scheduled follow-up visit were considered as lost to follow-up.

### Statistical Analysis

To obtain multiple ACR cut-off values that distinguished among distinct frequencies of incident macroalbuminuria, ROC curve analysis was performed using baseline ACR and the occurrence of macroalbuminuria. The Kaplan-Meier method and log-rank test were used to compare ACR categories assorted by cut-off points. Dichotomous variables were compared with the chi-square test. All pairwise comparisons were performed using ANOVA. A P value < 0.05 was considered statistically significant. All statistical analyses were performed using GraphPad Prism 5.02 software (GraphPad Software Inc. San Diego, CA).

## Additional Information

**How to cite this article**: Chida, S. *et al*. Levels of albuminuria and risk of developing macroalbuminuria in type 2 diabetes: historical cohort study. *Sci. Rep.* **6**, 26380; doi: 10.1038/srep26380 (2016).

## Figures and Tables

**Table 1:** Clinical characteristics of the 1760 patients stratified by baseline ACR categories.

Characteristic	ACR (mg albumin/gCr)				P value
	<7.5	≥7.5 and <30	≥30 and <150	≥150 and <300	
	(N = 533)	(N = 712)	(N = 469)	(N = 46)	
Age, yr	58.1 ± 12.0	61.6 ± 11.2	62.3 ± 11.4	62.0 ± 11.7	<0.0001¶
Male sex, no (%)	358 (67.2)	415 (58.3)	264 (56.3)	25 (54.3)	0.0014¶¶
Body mass index, kg/m^2^	23.2 ± 3.0	23.3 ± 3.1	23.6 ± 3.3	23.8 ± 3.4	0.27¶
Glycosylated haemoglobin, %	7.9 ± 1.7	8.4 ± 1.9	8.7 ± 1.8	8.5 ± 2.4	<0.0001¶
Blood pressure, mmHg					
Systolic	124 ± 16	129 ± 16	131 ± 16	141 ± 14	<0.0001¶
Diastolic	73 ± 10	75 ± 11	75 ± 11	80 ± 12	0.0008¶
Urinary albumin-to-creatinine ratio‡‡					
Median	4.5	13.6	54.4	180	ND
Interquartile range	3.0–5.8	10.0–19.1	38.1–82.1	165.4–220.5	
Serum creatinine, mg/dl‡					
Male patients	0.8 ± 0.1	0.8 ± 0.1	0.8 ± 0.1	0.8 ± 0.1	0.27¶
Female patients	0.6 ± 0.1	0.6 ± 0.1	0.6 ± 0.1	0.6 ± 0.1	0.41¶
Triglycerides, mg/dl**	143 ± 104	155 ± 105	172 ± 128	168 ± 109	0.0008¶
Cholesterol, mg/dl††					
Total	205 ± 40	207 ± 39	209 ± 44	206 ± 36	0.37¶
Low-density lipoprotein	128 ± 38	129 ± 38	129 ± 40	129 ± 31	0.99^¶^
High-density lipoprotein	59 ± 16	58 ± 16	57 ± 17	56 ± 16	0.25¶
TREATMENT					
Glucose lowering, no (%)					
Sulfonylurea	163 (30.6)	251 (35.3)	148 (31.6)	20 (43.5)	0.12¶¶
Glinides	20 (3.8)	18 (2.5)	12 (2.6)	2 (4.3)	0.53¶¶
Biguanide	88 (16.5)	115 (16.2)	79 (16.8)	4 (8.7)	0.56¶¶
Thiazolidinediones	34 (6.4)	30 (4.2)	25 (5.3)	4 (8.7)	0.26¶¶
Alpha glucosidase inhibitors	73 (13.7)	111 (15.6)	64 (13.6)	6 (13.0)	0.71¶¶
DPP-IV inhibitors	34 (6.4)	24 (3.4)	11 (2.3)	2 (4.3)	0.01¶¶
Insulin	164 (30.8)	204 (28.7)	151 (32.2)	8 (17.4)	0.15¶¶
Antihypertensive agents, no (%)					
Diuretics	24 (4.5)	40 (5.6)	38 (8.1)	3 (6.5)	0.11¶¶
Aldosterone antagonist	0 (0)	1 (0.1)	1 (0.2)	0 (0)	0.77¶¶
Alpha-blockers	12 (2.3)	31 (4.4)	23 (4.9)	1 (2.2)	0.11¶¶
Beta-blockers	20 (3.8)	32 (4.5)	34 (7.2)	1 (2.2)	0.047¶¶
Calcium-channel blockers	55 (10.3)	110 (15.4)	102 (21.7)	9 (19.6)	<0.0001¶¶
Dihydropyridines	48 (9.0)	104 (14.6)	97 (20.7)	9 (19.6)	<0.0001¶¶
Centrally acting agents	0 (0)	1 (0.1)	1 (0.2)	0 (0)	0.77¶¶
Angiotensin-I-converting enzyme inhibitors	15 (2.8)	50 (7.0)	41 (8.7)	5 (10.9)	0.0005¶¶
Angiotensin II receptor antagonists	61 (11.4)	109 (15.3)	96 (20.5)	8 (17.4)	0.0014¶¶
Lipid-lowering agents, no (%)					
Statins	82 (15.4)	114 (16.0)	88 (18.8)	5 (10.9)	0.33¶¶
Fibrates	10 (1.9)	14 (2.0)	17 (3.6)	3 (6.5)	0.07¶¶
Aspirin, no (%)	38 (7.1)	65 (9.1)	49 (10.4)	0 (0)	0.045¶¶

^*^Mean ± SD.

^‡^To convert values to micromoles per liter, multiply by 88.4.

^**^To convert values to millimoles per liter, multiply by 0.0113.

^††^To convert values to millimoles per liter, multiply by 0.0259.

^¶^Exploratory comparisons using ANOVA tests.

^¶¶^Exploratory comparisons using chi-square tests. ND, not done.

^‡‡^Albumin was measured in milligrams, and creatinine in grams. The baseline urinary albumin-to-creatinine ratio was defined as the mean of the first two consecutive measurements.
